# Ostéochondrome solitaire de la symphyse pubienne de découverte fortuite

**DOI:** 10.11604/pamj.2019.32.74.18076

**Published:** 2019-02-12

**Authors:** Dhouha Bacha, Asma Sassi, Sana Ben Slama, Mouadh Nefiss, Lassad Gharbi, Saadia Bouraoui, Ahlem Lahmar

**Affiliations:** 1 Service d’Anatomie et de Cytologie Pathologiques, Hôpital Mongi Slim, La Marsa, Tunis, Tunisie; 2 Service de Chirurgie Viscérale et Orthopédique, Hôpital Mongi Slim, La Marsa, Tunis, Tunisie

**Keywords:** Tissu osseux, ostéochondrome, cartilage, tumeur, symphyse pubienne, Bone tissue, osteochondroma, cartilage, neoplasm, pubic symphysis

## Abstract

L'ostéochondrome est la tumeur osseuse bénigne la plus fréquente. Elle touche habituellement les métaphyses des os longs, particulièrement autour du genou et de l'humérus proximal. Il touche très rarement la symphyse pubienne avec fréquemment une symptomatologie atypique. Nous rapportons le cas d'un ostéochondrome de la symphyse pubienne empiétant sur la branche osseuse ilio-pubienne chez un homme de 35 ans, de découverte fortuite. Les explorations radiologiques, l'examen macroscopique et histologique confirment le diagnostic ainsi que l'absence de signe de malignité.

## Abstract (en)

Osteochondroma is the most common benign bone tumor. It usually affects the metaphyses of the long bones, in particular around the knee and the proximal humerus. It very rarely affects the pubic symphysis, usually with an atypical symptomatology. We here report the case of a 35-year old man in whom osteochondroma in the pubic symphysis encroaching on the iliopubic branch was fortuitously discovered. Radiological examinations as well as macroscopic and histological examination confirmed the diagnosis and the absence of signs of malignancy.

## Introduction

L'ostéochondrome (OC) ou l'exostose ostéogénique est la plus fréquente des tumeurs osseuses, représentant 40% des tumeurs bénignes et 12% des tumeurs osseuses primitives [1, 2]. Il touche les sujets jeunes dans les 3 premières décades de la vie, avec une prédominance masculine (sex ratio homme/femme égal à 2) [3]. Il est le plus souvent solitaire mais parfois multiple dans la maladie des exostoses multiples, à transmission autosomique dominante. Son siège de prédilection est la métaphyse des os longs du fémur distal, de l'humérus proximal, du tibia proximal et du péroné. Il atteint le pelvis dans seulement 5 à 7% des cas, plus souvent la crête iliaque que les autres portions. Il touche très rarement la symphyse pubienne [3, 4]. Nous rapportons un cas d'OC de la symphyse pubienne gauche de découverte fortuite.

## Patient et observation

**Figure 1:**
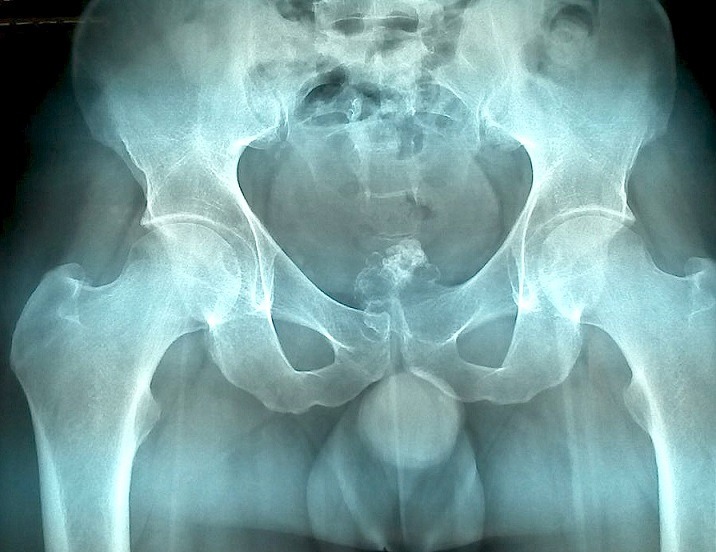
Radiographie du bassin: processus pédiculé en regard de la symphyse pubienne gauche, empiétant sur la branche osseuse ilio-pubienne, en «chou-fleur», d’allure hétérogène, mal limité et calcifié

**Figure 2:**
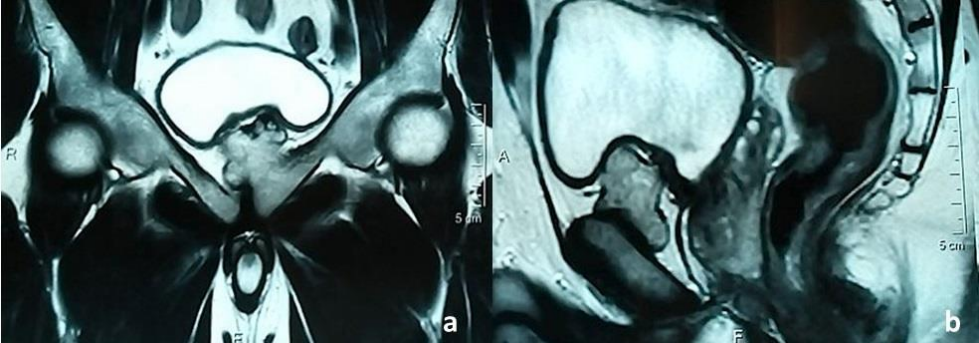
IRM du bassin: excroissance osseuse de la symphyse pubienne gauche, à large base d’implantation, lobulée dans sa partie distale et laminant la vessie qui est homogène et à paroi fine: (a) coupe frontale; (b) coupe sagittale

Il s'agit d'un homme âgé de 35 ans, sans antécédents particuliers, suivi depuis 6 mois en rhumatologie pour des polyarthralgies chroniques de type inflammatoire, migratrices, touchant les grosses articulations des membres. Il a été mis sous traitement anti-inflammatoire. L'examen physique ne trouvait pas d'anomalies, en particuliers celles de la marche et de l'appui. A la radiographie du bassin, il a été découvert fortuitement une excroissance osseuse de la symphyse pubienne empiétant sur la branche iléo-pubienne gauche (Figure 1). L'imagerie par résonnance magnétique (IRM) du pubis avait montré que cette excroissance était pédiculée, à base d'implantation large, présentant une corticale et un os spongieux en continuité avec l'os porteur. La masse était lobulée dans sa portion distale. Elle exerçait un effet de masse sur la vessie qui était par ailleurs de plage homogène et à paroi fine. L'excroissance osseuse était munie d'une fine coiffe cartilagineuse dont l'épaisseur ne dépassait pas 4 mm, sans anomalies du signal et sans réhaussement pathologique (Figure 2). Cet aspect était en faveur d'un OC sans signe de dégénérescence.

**Figure 3:**
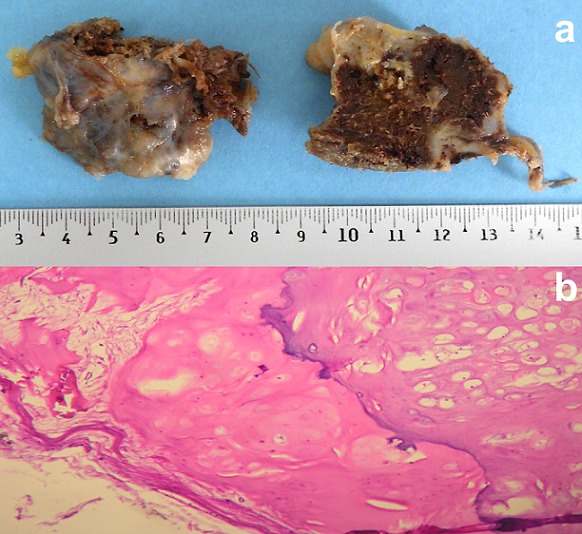
Aspect anatomo-pathologique: (a) macroscopie: pièce osseuse surmontée par un tissu cartilagineux plus ou moins régulier; (b) histologie: capsule fibreuse recouvrant une coiffe cartilagineuse peu cellulaire qui s’ossifie en profondeur (hématoxylline éosine x 200)

Le patient a eu une ostéotomie en-bloc de la tumeur au niveau de sa base. La pièce osseuse mesurait 4,5x3,5 cm et était surmontée d'un cartilage plus ou moins régulier (Figure 3). L'examen histologique après décalcification préalable trouvait, sous une capsule fibreuse, une coiffe cartilagineuse à cellularité peu dense avec généralement un seul élément cellulaire par cavité chondroblastique. Le cartilage s'ossifie en profondeur pour produire un os spongieux normal. En périphérie, des travées osseuses pré-existantes délimitaient des espaces hématopoïétiques de morphologie habituelle. Il n'a pas été noté de signe de malignité (Figure 3). Ces aspects confirmaient le diagnostic d'ostéochondrome sans signe de dégénérescence. Deux ans après la chirurgie, le patient ne présentait pas de signe de récidive ou de dégénérescence.

## Discussion

L'OC est souvent asymptomatique. Il devient symptomatique lorsqu'il est de grande taille ou de siège anatomique critique comme celui du pelvis [2, 5]. Dans ce cas, il se manifeste souvent par des signes atypiques, témoins de compression nerveuse, vasculaire, uréthrale ou musculo-squelettique des organes adjacents [2]. En effet, il a été rapporté des cas d'OC de la symphyse pubienne avec des perturbations sexuelles [2, 5, 6], une masse prostatique [7] ou une compression vésicale [8] et des cas d'OC de l'ischion avec des sciatalgies [1, 3, 9]. Ceci implique plusieurs spécialités comme l'urologie, la gynécologie et la rhumatologie dont les spécialistes doivent évoquer une anomalie osseuse de la région pubienne comme origine de ces symptômes. Dans notre cas, la découverte était fortuite suite à un bilan radiologique pour des arthralgies chroniques. L'exploration radiologique est capitale pour évoquer le diagnostic d'une part et pour décider de l'acte chirurgical d'autre part. La TDM est utile dans les OC de localisations atypiques comme dans notre cas. Elle permet de définir l'intégrité de la zone corticale et la distribution des calcifications [3]. L'IRM permet de planifier la technique chirurgicale puisqu'elle permet d'évaluer l'atteinte intra-osseuse et des tissus mous adjacents à la tumeur [10]. L'IRM permet également de suspecter une dégénérescence de l'OC par la mesure de la coiffe cartilagineuse qui doit être inférieure à 2 cm d'épaisseur. Les examens radiologiques révèlent une excroissance osseuse sessile ou pédiculée. L'os cortical de l'exostose est en continuité avec la corticale de l'os porteur et l'os spongieux au sein de l'exostose et en continuité avec le spongieux sous-jacent. Etant donné que la coiffe cartilagineuse est non radio-opaque, seuls les éventuels foyers de calcifications de celle-ci sont visibles. Ces calcifications sont d'aspect cotonneux dans l'OC [9, 10].

Le rôle du pathologiste consiste dans la majorité des cas à confirmer le diagnostic d'OC. Il a une architecture caractéristique associant de dehors en dedans, une fine lame fibreuse, une coiffe cartilagineuse et un tissu osseux d'architecture trabéculaire constituant le corps de l'exostose. L'aspect histopathologique de la coiffe cartilagineuse est très proche de celui d'un cartilage de conjugaison, avec des chondrocytes regroupés en îlots en surface, en colonne en profondeur. Ces chondrocytes ne présentent pas d'atypies marquées. En profondeur, ce cartilage donne naissance à un os mature par ossification enchondrale. Ce processus d'ossification enchondrale est parfois incomplet, laissant persister de petites plages de cartilage au sein du corps osseux de l'OC. Ce cartilage va se calcifier, puis se nécroser [3, 9]. L'OC pelvien pose le problème du diagnostic différentiel avec le chondrosarcome et la myosite ossifiante [3]. La possibilité d'une transformation chondrosarcomateuse d'un OC, même si elle reste très faible, devra être éliminée. Elle est plus fréquente dans la maladie des exostoses multiples (3 à 5% contre 1% si l'OC est solitaire) [5, 9]. Dans la symphyse pubienne, son développement est souvent lent, ce qui peut être parfois initialement considéré, de ce fait, comme des tumeurs bénignes [11]. Les chondrosarcomes développés sur un OC sont algiques même en l'absence de fracture, de bursite ou de compression nerveuse. Sur le plan radiologique, une poussée évolutive de l'OC, le développement d'une volumineuse coiffe cartilagineuse avec des calcifications dispersées et une infiltration des tissus mous adjacents sont également des signes en faveur de la dégénérescence [3, 10]. De plus, il existe une alternance de zones de résorption endostéale cupuliforme, étendues et profondes et des zones d'épaississement et d'ostéosclérose corticale [3]. La scintigraphie n'est pas d'un apport diagnostique dans ce cadre [3, 10]. Les chondrosarcomes développés sur un OC sont des tumeurs très bien différenciées, de bas grade, avec un diagnostic difficile, ne pouvant être fait que sur la pièce d'exérèse [12].

Sur le plan histologique, les chondrosarcomes conservent un aspect cytologique très proche de celui du cartilage constituant la coiffe de l'OC et leur diagnostic va reposer avant tout sur des critères architecturaux. Une transformation devra être suspectée devant toute modification de l'architecture de l'OC et plus particulièrement de sa coiffe. Cette dernière devient irrégulière et lobulée par des septa fibreux. Des lobules cartilagineux diffusent dans les tissus mous adjacents. D'autres critères histologiques comme la présence de remaniements myxoïdes et d'atypies nucléaires des chondrocytes devront être recherchés mais sont en pratique rarement observés [12]. Dans notre cas, la coiffe cartilagineuse était fine et régulière, sans anomalies du signal et sans réhaussement pathologique et sur le plan histologique, elle était peu cellulaire. Concernant la myosite ossifiante, il s'agit d'un processus bénin d'ossification hétérotopique focale des parties molles, survenant chez le sujet jeune le plus souvent à la suite d'un traumatisme [13]. Sur le plan de l'imagerie, à la phase de maturation, apparaissent des calcifications en couronne qui s'organisent autour d'un centre clair. La persistance d'une zone de transparence normale entre la lésion et la corticale osseuse adjacente doit permettre d'orienter le diagnostic vers une lésion bénigne [13]. A la phase chronique, la tomodensitométrie (TDM) met en évidence l'aspect caractéristique de coque osseuse compacte. Aucune excroissance osseuse bourgeonnante surmontée d'une coiffe cartilagineuse n'existe autour de celle-ci, comme c'est le cas dans notre observation. L'IRM, lorsqu'elle est réalisable, permet de déceler encore plus précocement la myosite avant l'apparition de calcifications [13].

L'indication chirurgicale n'est pas absolue en cas d'OC pelvien. Elle est surtout discutée en cas de complications ou de tumeur pédiculée [14]. Dans ce cas, le traitement de choix est une ostéotomie en-bloc de la lésion selon une approche transversale centrée sur l'OC [2]. Cette résection doit être complète puisque la persistance de périchondre ou de coiffe cartilagineuse serait prédictif de récidive locale de l'OC [9, 14]. Certaines précautions chirurgicales doivent être considérées, comme la préservation du cordon inguinal, de l'urètre et de la bande neuro-vasculaire obturatrice [2]. Dans notre cas, malgré le caractère asymptomatique de l'OC, une chirurgie a été indiquée du fait du caractère pédiculé de la tumeur et de son contact étroit avec la vessie, ce qui pourrait générer des complications urologiques. Une surveillance radio-clinique étroite doit être établie pendant les 2 premières années après la chirurgie à la recherche d'une récidive locale de la tumeur ou de signe de dégénérescence [14]. Dans notre cas, après 2 ans d'évolution, il n'a pas été noté de signe de récidive ou de dégénérescence.

## Conclusion

L'OC de la symphyse pubienne est très rare. Sa symptomatologie est souvent atypique, touchant différentes spécialités comme l'urologie, la gynécologie ou la rhumatologie. Il peut être de découverte fortuite. Le bilan radiologique est capital pour évoquer le diagnostic et pour la conduite thérapeutique. La résection tumorale en-bloc est le traitement de choix pour les OC pédiculés et ceux symptomatiques. Le pathologiste confirme le diagnostic et élimine en priorité une dégénérescence chondrosarcomateuse. Une surveillance radio-clinique étroite est conseillée les 2 premières années après la chirurgie pour détecter une éventuelle récidive locale ou une dégénérescence.

## Conflits d’intérêts

Les auteurs ne déclarent aucun conflit d'intérêts.
